# Legalization of marijuana or not? Opinions from over 38,000 residents in Taiwan

**DOI:** 10.1186/s12889-023-16834-x

**Published:** 2023-10-09

**Authors:** Kuo-Yu Chao, Shu-Hsiang Liu, Chih-Chiang Chou, Ching-I Chen, Wei Cheng

**Affiliations:** 1 Department of Nursing, Chang Gung University of Science and Technology, Taoyuan, Taiwan; 2 Division of Colon and Rectal Surgery, Chang Gung Memorial Hospital, Taoyuan, Taiwan; 3 School of Nursing, National Taipei University of Nursing and Health Sciences, Taipei, Taiwan; 4 Department of Psychiatry, Centro Hospitalar Conde de São Januário, Macau SAR, China; 5 Department of Psychiatry, Kee-Lung Hospital, Ministry of Health and Welfare, KeeLung, Taiwan; 6 Department of Pathology, Kee-Lung Hospital, Ministry of Health and Welfare, 268, Xin 2nd Road, Xinyi District, KeeLung, 201203 Taiwan; 7 Department of Nursing, Deh Yu College of Nursing and Health, Kee-Lung, Taiwan

**Keywords:** Marijuana, Cannabis, Medical marijuana, Tetrahydrocannabinol (THC), Cannabidiol (CBD), Legalization

## Abstract

**Background:**

Marijuana is legal in many Western countries and Thailand. In Taiwan, Marijuana remains a category-2 narcotic; however, some legislative candidates recently advocated legalization of medical marijuana. This study surveyed a large sample of Taiwanese to gain a better understanding of the public’s knowledge and attitudes towards legalizing marijuana.

**Methods:**

This cross-sectional mixed-methods study included demographic data and responses to a survey questionnaire, “Knowledge and Attitudes of Legalizing Marijuana” (KALM). The survey included 15 statements about four categories: public health, social impact, medical applications of THC (Δ^9^-tetrahydrocannabinol), and legal and tax consequences; and two yes/no questions about medical use and legalization of marijuana. Knowledge was scored as disagree = 0, no knowledge = 2, or agree = 4; attitude was scored from 0 = very unimportant to 4 = very important. Responses to an open-ended question asking for additional comments/concerns were analysed with content analysis. The survey was conducted from February 15 to March 1, 2023.

**Results:**

Data were analysed from 38,502 respondents, aged 15 to > 56 years. Most were female (67.1%) and parents (76.4%). Scores were higher for respondents who were parents, religious, ≥ 36 years of age, had a high-income status, no history of substance abuse, knowledge of medical marijuana, and did not support legalization of marijuana. Medical personnel had greater knowledge of marijuana, but their attitude indicated they viewed legalization as less important. In the open-ended question, many respondents requested more information about marijuana be provided to the public before considering legalization.

**Conclusions:**

Taiwanese respondents considered legalization of marijuana a significant concern, especially as it relates to impacts on public health.

**Supplementary Information:**

The online version contains supplementary material available at 10.1186/s12889-023-16834-x.

## Introduction

The evolution of policies legalizing marijuana in states in the US has prompted similar policy changes in other countries [1]. Marijuana policy in North America has shifted since the 1990s with legalization of medical marijuana in some states in the US and in Canada in 2018 followed by legalization of large-scale commercial marijuana production and sale of marijuana for recreational use. Legalization of marijuana has spread globally; in Taiwan, legalization has been promoted by some legislators during recent elections and advocated by some groups [2].

Legalization of marijuana for recreational use initially occurred by popular vote in the states of Colorado and Washington in 2012 in the USA. As of April 20, 2023, 20 states and the District of Columbia have legalized marijuana for recreational use while 27 states allow it for various medicinal purposes, although marijuana remains illegal under US Federal law [3], despite possible benefits and potential therapeutic uses [1]. However, following legalization of recreational marijuana in Canada, the frequency and severity of children admitted to the emergency department increased significantly due to marijuana exposure [4]. Thailand also reported children experienced harmful effects after consuming “edible” marijuana, products sold as chocolates or candy, following legalization for medical and commercial use [5].

Drug misuse is one of the major social, legal, and public-health challenges in the modern world, which includes child and adolescent misuse and abuse. In the United States, one of the most abused substances reported by adolescents is marijuana (1.1% among 8th graders, 4.4% among 10th graders, and 6.9% among 12th graders 1n 2020) [6]. Although illegal, abuse of marijuana is also high in Taiwan (2.6% in ≦ 19 years old illicit drug users in 2019) [7].

Historically, medicinal use of marijuana has preceded recreational use, which was initially permitted for a short list of medical conditions (nausea, weight loss, pain, muscle spasm, and serious medical conditions) [1]. Qualifications were progressively broadened, which enabled adults to obtain a medical recommendation and purchase marijuana from retail dispensaries [1]. Countries in Europe, Oceania, Africa, and Asia have since permitted the use of medical marijuana [1]. The term “medical marijuana” refers to using the whole, unprocessed marijuana plant or its extracts [8]. Marijuana-based medications, such as THC (Δ^9^-tetrahydrocannabinol) and CBD (cannabidiol) undergoing clinical trials have been demonstrated to be effective in some diseases [8]. The addition of FDA approved CBD to anti-epileptic drugs reduced the frequency of epileptic seizures in children with Dravet and Lennox-Gastaut syndromes [9] and THC was shown to be more effective than placebo in reducing nausea and vomiting associated with cancer chemotherapy [10]. However, the Food and Drug Administration (FDA) in the US does not recognize, regulate, or approve of marijuana as a medicine as it can result in adverse health effects from smoking and cognitive impairment from THC [8]. Controversies remain surrounding safe administration, packaging, and dispensing of medical marijuana [11].

Surveys conducted with residents of the state of Vermont in the US about marijuana have shifted in favour of legalization [12], and support for legalizing marijuana continues to rise in other areas of the United States [13]. Calls to legalize marijuana are mounting across Europe, as a growing number of countries seek this progressive move [14]. However, in Taiwan, marijuana remains a category-2 narcotic. In 2021, many legislative candidates in Taiwan advocated legalization of medical marijuana and subsequent legalization of recreational marijuana. One of these supporters, Bai-Wei Chen, won election as a legislator [2].

Due to the pending policy change in Taiwan regarding medical and recreational use of marijuana, this study surveyed residents of Taiwan to gain an understanding of knowledge and attitudes towards legalization of marijuana. An online survey was designed and disseminated across Taiwan and the Taiwanese islands. Responses to the survey questionnaire and demographic data examined if any survey categories were associated with characteristics of the respondents.

## Methods

### Design

This cross-sectional study examined knowledge about and attitudes towards legalization of marijuana based on an online survey disseminated by non-government organizations comprised of parents, teachers, students, and physicians and distributed across Taiwan. The use of the term “marijuana” throughout this article represents the cannabis plant and its extracts. Data were collected between February 15 and March 1, 2023.

### Participants

Residents of Taiwan (aged 15 years and above) were invited to participate in the online survey through a link provided by a national organization of public affairs and non-governmental agencies in Taiwan. Ethical approval for this study was obtained from the Ethics Committee of Taipei Hospital, Ministry of Health and Welfare (TH-IRB-0022-0029). All procedures performed were in accordance with the Ethics Committee and the 1964 Helsinki declaration and its later amendments or comparable ethical standards. Though we included participants aged 15–19 years, the Ethics Committee approved our request to waive the documentation of informed consent because the online survey was anonymous and had minimized possible harms. Sample size was calculated for a precision level of ± 0.5%, a confidence interval of 95% and *p* = 0.5 (maximum variability), which was determined to be 38,416 [15]. Considering a 1% rate of attrition, 38,800 valid surveys were needed.

### The survey

We developed a self-report survey to examine knowledge of marijuana usage and attitudes towards legalizing marijuana (KALM). The first part of the survey collected data on demographic characteristics and the respondent’s views of whether there was valid, reliable evidence supporting for the use of medical marijuana (yes/no) and whether marijuana use should be legalized (yes/no).

The second part of the KALM survey was comprised of 15 statements about marijuana which were guided by reports by various government agencies and published meta-analyses and systemic reviews. We did not include any questions pertaining to the therapeutic efficacy of marijuana because results are mixed and there is a paucity of valid, reliable, and empirical evidence the application of marijuana or medical marijuana, which require large-scale clinical trials [16, 17]. We also did not included questions on the use of CBD (cannabidiol) because it can be confused with marijuana [16].

The survey was divided into four categories for knowledge and attitudes: public health (4 items), such as “Marijuana use during pregnancy may cause stillbirths, preterm births and long-term brain defects” [18]; social impact (5 items), such as “Marijuana use is associated with a 5- to 10-fold increase in violence” [19, 20]; medicine and use of THC (3 items), such as “Increased paranoia, psychosis and addiction are more prevalent with consumption of high concentrations of THC” [21]; and legal and tax consequences (3 items), such as “People spend $4.50 for every $1 in tax revenue that legal marijuana generates” [22]. We attempted to balance positive and negative perspectives for each category, however, there is little research data on positive social impacts of nonmedical marijuana use [23]. The 15 statements and the supporting citations used to develop the statements are shown in Table 1. Knowledge of each statement was assessed with three possible responses: agree (4 points), no knowledge (2 points) and disagree (0 points). Three statements (4, 12, and 13) were reverse scored, with agree, no knowledge, and disagree scored as 0, 2 and 4, respectively. Attitude was scored on a 5-point Likert scale from 0 = very unimportant to 4 = very important. Therefore, the total scores for both knowledge and attitude on the KALM survey ranged from 0 to 60. Higher scores for knowledge indicated participants had more accurate information about marijuana; higher scores for attitude indicated a participants viewed the statement as having a greater impact on Taiwanese society.

**Table 1:** Survey responses for Knowledge and Attitude of Legalizing Marijuana (KALM) (N = 38,502)

		Frequency of responses						Reference#
		Agree	No knowledge	Disagree	Knowledge^**+**^		Attitude^‡^	
Items	Categories and items (statements)	n (%)	n (%)	n (%)	Mean ± SD		Mean ± SD	
Public Health (range = 0 to 16)								
1.	Marijuana use during pregnancy may cause stillbirths, preterm births, and long-term brain defects	34,037 (88.4%)	1,699 (4.4%)	2,766 (7.2%)	3.62 ± 1.09		3.71 ± 0.93	[18]
2.	Persistent use of marijuana during onset of adolescence causes a loss of IQ, which is not fully restored by cessation of marijuana use.	35,218 (91.5%)	1,555 (4.0%)	1,729 (4.5%)	3.74 ± 0.90		3.72 ± 0.89	[35]
3.	Emergency room records in Colorado show a several-fold increase in marijuana cases since legalization of sales of recreational marijuana.	28,863 (75%)	7,163 (18.6%)	2,476 (6.4%)	3.37 ± 1.17		3.65 ± 0.92	[42]
4.	Marijuana is not addictive. ^¶^	5,595 (14.5%)	1,376 (3.6%)	31,531 (81.9)	3.35 ± 1.43		3.70 ± 0.88	[43]
	**Category score, range = 0 to 16**				**14.08 ± 3.21**		**14.77 ± 3.31**	
Social Impact (range = 0 to 20)								
5.	Marijuana use is associated with a 5- to 10-fold increase in violence.	33,250 (86.4%)	3,459 (9.0%)	1,793 (4.7%)	3.63 ± 0.99		3.70 ± 0.87	[19, 20]
6.	Marijuana use increases the risk of car accidents as much as 14-fold and doubles the odds of a fatal collision	32,683 (84.9%)	4,231 (11.0%)	1,588 (4.6%)	3.62 ± 0 0.97		3.71 ± 0.86	[44, 45]
7.	Drug-related school suspension rates have risen in high schools where legalization has occurred.	30,923 (80.3%)	5,801 (15.1%)	1,778 (4.6%)	3.51 ± 1.05		3.68 ± 0.89	[46]
8.	Marijuana-related deaths involving car accidents, fire fatalities, explosions, and homicides increased in Colorado since legalization.	30,643 (79.6%)	6,035 (15.7%)	1,824 (4.7%)	3.50 ± 1.06		3.68 ± 0.88	[47]
9.	Marijuana is a “gateway drug” —using marijuana increases the risk of using other drugs.	34,613 (89.9%)	1,517 (3.9%)	2,372 (6.2%)	3.67 ± 1.02		3.71 ± 0.87	[48]
	**Category score, range = 0 to 20**				**17.93 ± 4.53**		**18.48 ± 4.19**	
Medicine and use of THC/marijuana, (range = 0 to 12)								
10.	Every state that has legalized recreational marijuana legalized medical marijuana first.	18,828 (48.9%)	11,500 (29.9%)	8,174 (21.2%)	2.55 ± 1.58		3.57 ± 0.91	[1]
11.	Increased paranoia, psychosis and addiction are more prevalent with consumption of high concentrations of THC.	32,208 (83.7%)	4,653 (12.1%)	1,641 (4.3%)	3.59 ± 1.00		3.68 ± 0.85	[21]
12.	Overuse of marijuana is harmless.^¶^	4,226 (11.0%)	906 (2.4%)	33,370 (86.7%)	3.51 ± 1.27		3.71 ± 0.84	[21, 35]
	**Category score, range = 0 to12**				**9.66 ± 2.41**		**10.96 ± 2.39**	
Legal and Tax Consequences (range = 0 to 12)								
13.	Originally, punishment for growing marijuana for personal use was a minimum 5-year fixed-term imprisonment; now it is a 1-year minimum to a 7-year maximum in Taiwan. ^¶,¥^	5,097 (13.2%)	5,783 (15.0%)	27,622 (71.7%)	3.17 ± 1.42		3.68 ± 0.83	[49, 50]
14.	Although minors cannot legally use marijuana, over 1/3 of US high school students report lifetime use of marijuana.	17,772 (46.2%)	20,730 (53.8%)	N/A^§^	1.85 ± 2.00		3.63 ± 0.88	[51]
15.	People spend $4.5 for every $1 in tax revenue that legal marijuana generates.	22,660 (58.9%)	11,681 (30.3%)	4,161 (10.8%)	2.96 ± 1.36		3.63 ± 0.88	[22]
	**Category score, range = 0 to12**				**7.98 ± 3.18**		**10.94 ± 2.43**	
**Total KALM score**,** range = 0 to 60**					**49.65 ± 10.33**		**55.15 ± 11.77**	

SD = standard deviation; THC = Δ^9^-tetrahydrocannabinol

^**+**^ Knowledge of each statement was scored as Agree (4 pts), No Knowledge (2 pts), or Disagree (0 pts). The total category score was the sum of the item scores divided by the number of items in the category

^‡^ Attitude towards each statement was scored on a 5-point Likert scale from 0 (very unimportant) to 4 (very important). The total score for each category was the sum of the item scores divided by the total number of items

^¶^ Indicates the question was reverse scored: Disagree (4 pts) + No Knowledge (2 pts) + Agree (0 pts)

^¥^ Reduced from a felony with of term of ≥ 5 years to a misdemeanor, which can be suspended or pardoned

(1) Persons guilty of possession with intention to sell Cannabis shall be punished with a minimum 5-year fixed-term imprisonment, and may be fined of no more than 5 million New Taiwan dollars (NTD).

(2) Persons convicted of cultivating Cannabis with intentions to supply for manufacturing narcotics shall be punished with a minimum 5-year fixed-term imprisonment, and may be fined no more than 5 million NTD. Persons convicted of cultivating Cannabis for personal use shall be punished with a minimum 1-year to a maximum 7-year fixed-term imprisonment; and in addition thereto, a fine of not more than 1 million NTD may be imposed

^§^ Choices were limited to ‘agree’ or ‘no acknowledge’ because this was based on data from the CDC.

Reference # = supporting citation for the statement

Because the survey population was to include adolescents aged > 15 years, face validity of statements was assessed for readability and comprehension by one senior high school student and three junior high school students, comprised of two boys and two girls. Each statement was evaluated on a 5-point Likert scale from 1 = difficult to read or understand to 5 = highly readable and understandable. The mean total score for face validity was 4.58, indicating no changes were needed in the wording of the statements.

Content validity of the 15 items of the KALM survey was examined by a panel of four experts in the fields related to marijuana: one psychologist with PhD; a pharmacist with master’s degree; a lawyer with master’s degree, and a medical technician. All experts rated each item for relevance using a 5-point Likert scale (1 = not relevant to 5 = very relevant) and the content validity index (CVI) was calculated. The CVI is computed as the number of experts giving a rating of “very relevant” for each item divided by the total number of experts. A CVI > 0.79 indicates the item is relevant, between 0.70 and 0.79, the item needs revisions, and if the value is below 0.70 the item is deleted. None of the items were revised or deleted and all items received a rating ≥ 4, indicating good content validity according to Lynn (1986) [24]. The content validity index (CVI) was 98%, indicating good validity of the survey items.

Cronbach’s alpha for internal consistency of the four survey categories was 0.935 (public health), 0.979 (social impact), 0.908 (medicine and use of THC), and 0.972 (legal and tax consequences). The Cronbach’s alpha for the total score on the KALM survey was 0.982. The fit indices following confirmatory factor analysis were > 0.50 for Average Variance Extracted; 0.939 for the goodness of fit index (GFI); 0.904 for adjusted GFI, and the relative fit indices (normed FI (NFI) and parsimonious NFI were over 0.9 [25]. Therefore, the survey had good reliability and validity and could serve as a tool for collecting data about knowledge and attitudes of legalizing marijuana.

A third section of the survey allowed respondents to provide qualitative feedback about their opinions of legalization of marijuana. This was an open-ended question, asking whether there was anything they would like to share about legalization of marijuana.

### Data collection

Several non-government organizations comprised of parent groups, teachers, students, and physicians throughout Taiwan notified the public of the KALM survey through their social media sites; heads of the organizations provided a link to the site. Responders could fill out the survey only once, which was determined by their Internet Protocol Address (IP address) through SurveyCake, which allows for anonymous collection of data. If a questionnaire was not answered completely, it was failed data and could not be sent out. None of the surveys were distributed by mail, therefore only individuals with Internet access participated.

### Data analysis

Quantitative data were analysed using SPSS version 22.0 for Windows (Armonk, NY: IBM Corp). Characteristics of respondents were analysed with frequencies (n, %). Mean scores and the standard deviation (SD) were used for the total score on the KALM score as well as scores for statements and total scores for the four categories. Survey data from this large sample was assumed to be continuous, thus differences in mean scores for statements and total scores for the four categories of knowledge and attitudes were compared between groups of participants based on characteristics, which were analysed with independent t tests. Significance was set at *p* < 0.05 for statistical comparisons.

Qualitative data from the open-ended responses were collected and analysed with content analysis [26]. First, two researchers independently read all responses several times to gain a sense of the whole. Next, initial codes were generated from the responses. For instance, “Educating the public about other countries’ experiences of legalization of marijuana” was coded as “information about marijuana”. The researchers then searched for overall themes, such as “Promote accurate information about marijuana.” The two researchers then compared and discussed the themes until consensus was reached.

## Results

### Demographics of respondents

A total of 39,971 respondents submitted the online KALM survey. However, 43 respondents were under 15 years of age, and 938 surveys had a duplicate IP address, suggesting the same respondents completed the survey more than once. A total of 488 surveys had both agree and disagree options checked in the knowledge section, and the questionnaires were considered invalid. Thus, there was a sample loss of 3.68% and survey data were analysed from 38,502 respondents representing a broad spectrum of Taiwanese residents (Supplementary **Table S1**).

Characteristics of the respondents for the valid surveys are shown in Table 2. Most (76.4%) were parents and a small percent (6.8%, n = 2,622) were medical or healthcare personnel. A small percent of respondents were smokers (7.1%, n = 2,728), drank alcohol (9.4%, n = 3,603), or had used controlled drugs without a doctor’s order (1.9%, n = 747). 43.1% of respondents agree with the statement that there was a lack of valid, reliable, and empirical evidence to support the use of medical marijuana (n = 16,604) and only 5.1% (n = 1,970) agreed with legalizing marijuana.

**Table 2:** Characteristics of participants responding to the survey on Knowledge and Attitude of Legalizing Marijuana (KALM) (N = 38,502)

Characteristic	n	%
Gender		
Female	25,849	67.1%
Male	12,653	32.9%
Age, years^a^		
15–18	414	1.1%
19–24	886	2.3%
25–35	2,898	7.5%
36–45	6,472	16.8%
46–55	12,112	31.5%
≥ 56	15,720	40.8%
Parent		
Yes	29.443	76.4%
No	9,069	23.6%
Employed in medicine or healthcare		
Yes	2,622	6.8%
No	35,880	93.2%
Religious beliefs		
Yes	30,676	79.7%
No	7,826	20.3%
Low income or middle-low income		
Yes	5,035	13.1%
No	33,467	86.9%
Cigarette smoker		
Yes	2,728	7.1%
No	35,774	92.9%
Alcohol consumption		
Yes	3,603	9.4%
No	34,899	90.6%
Used controlled drugs without a prescription from a doctor		
Yes	747	1.9%
No	37,755	98.1%
There is a lack of valid, reliable evidence supporting marijuana (plants or extracts) as medically beneficial		
Agree	16,604	43.1%
Disagree	7,689	20.0%
No opinion	14,209	36.9%
Marijuana should be legalized		
Agree	1,970	5.1%
Disagree	36,011	93.5%
No opinion	521	1.4%

^a^Respondents were not asked to report their age; they only checked one of the six age ranges and there was no upper age limit

### Knowledge and attitudes of respondents

Frequencies and mean scores for the statements and mean total scores for each of the four categories of the KALM survey are shown in Table 1. Scores for the statements and the total score for the category of public health were similar for knowledge and attitudes. Most respondents agreed with the first three statements regarding the negative effects of marijuana on public health and 81.9% disagreed with the statement that marijuana was not addictive, which were appropriate responses. The total score for knowledge and attitudes ranged from 0 to 16 points; respondents scores were 14.08 (SD = 3.21) and 14.77 (SD = 3.31), respectively. These findings indicated respondents had a high level of knowledge about marijuana and strong attitudes about the impact of marijuana on public health.

Most respondents (79.6–89.9%) agreed with all five statements about the social impact of marijuana use. The mean total scores for knowledge and attitudes were 17.93 (SD = 4.53) and 18.48 (SD = 4.19), respectively. These scores indicate respondents were knowledgeable about the negative impact of marijuana on society; respondents also felt these negative aspects of marijuana use were important concerns.

For the category of medicine and the use of THC/marijuana, respondents had less knowledge regarding the order in which medical and recreational marijuana is legalized, with only 48.9% agreeing that typically medical marijuana is legalized first. However, most (83.7%) were aware that high concentrations of THC increased paranoia, psychosis, and addiction, and 86.7% disagreed with the statement that overuse of marijuana was harmless. The mean total score for knowledge was high (9.66, SD = 2.41), and the mean score for attitude (10.96, SD = 2.39) indicated respondents felt strongly that there were negative consequences of legalization of marijuana.

The last category of the KALM involved the legal and tax consequences of marijuana use. Most correctly disagreed (71.7%) with the statement regarding a reduction in punishment for the personal use of marijuana, from a 5-year minimum sentence to a 1-year minimum and a 7-year maximum. Most (53.8%) had no knowledge that over 1/3 of high school students reported lifetime use of marijuana, even though minors cannot legally use marijuana. Finally, most respondents (58.9%) were aware tax revenue generates only a small percentage of what is spent on legal purchases of marijuana. Although the total mean score for knowledge was lower than the total possible compared with the other three categories (7.98, SD = 3.18), total mean scores for attitude were high (10.94, SD = 2.43). These findings suggest the impact of legal and tax consequences of recreational marijuana use were importance concerns for the respondents.

The total possible score for overall knowledge and attitudes on the KALM survey was 60 points. The mean overall score for knowledge was 49.65 (SD = 10.33) and 55.15 (SD = 11.77) for attitude. Our findings suggest the Taiwanese survey respondents were well-informed about the 15 aspects of marijuana use presented in this survey and had strong concerns about marijuana’s impact.

### Knowledge and attitudes of marijuana by characteristics of respondents

We examined if the knowledge or attitude scores varied with any demographic characteristics of the respondents: parental status (yes/no), religious belief (yes/no), age ≤ 35 years vs. ≥ 36 years, low/middle to low income (yes/no), cigarette smoker (yes/no), alcohol consumption (yes/no), previous use of a controlled drug without a prescription (yes/no), medicine/health personnel (yes/no), and an opinion that marijuana should be legalized (yes/no) (Supplementary **Table S2**). For all four survey categories, scores for attitude differed significantly between groups (*p* < 0.01) for all demographic characteristics. For knowledge, two categories did not differ between respondents who were medicine/healthcare personnel compared with those who were not: public health knowledge (t = 1.23, *p* = 0.55) and knowledge of medicine and use of THC (t = 2.09, *p* = 0.31). In addition, overall total scores also differed significantly for all groups (all *p* < 0.01) (Supplementary **Table S3**).

For both mean category and overall total scores, when compared with their matched pair, scores were higher for respondents who were parents, with religious beliefs, ≥ 36 years of age, had a higher income, non-smokers, non-alcohol consumers, and non-drug users.

### Personal feedback about legalizing marijuana

Qualitative descriptive studies allow researchers to gain an understanding of the experiences of individuals which is closer to the truth [27]. Therefore, we collected responses to the open-ended question as anonymous feedback. Analysis of the comments revealed six themes: Promoting knowledge about marijuana; Personal, negative experiences of marijuana use; Use of marijuana abroad in regions where it is legal; Controversy of “medical” versus “recreational” marijuana and pharmaceutical standards; Economic reasons for not legalizing marijuana (harms outweigh benefits); and Support for legalization of marijuana.

Many respondents commented that the government should educate the public about what changes will occur if marijuana is legalized. Some respondents shared their negative experiences of using marijuana, such as not feeling well. Respondents who had lived abroad in countries where marijuana was legal talked about what life was like under these circumstances. Several respondents had questions about what the difference would be between legalizing “medical” versus “recreational marijuana”. A few respondents felt that the legalization of marijuana in Taiwan for monetary reasons was not a justification. However, there were respondents who did support legalizing marijuana due to the global trend in other countries. A description of the age and parental status of the respondents, and their comments are shown in Supplementary **Table S4**.

## Discussion

**Fig. 1:**
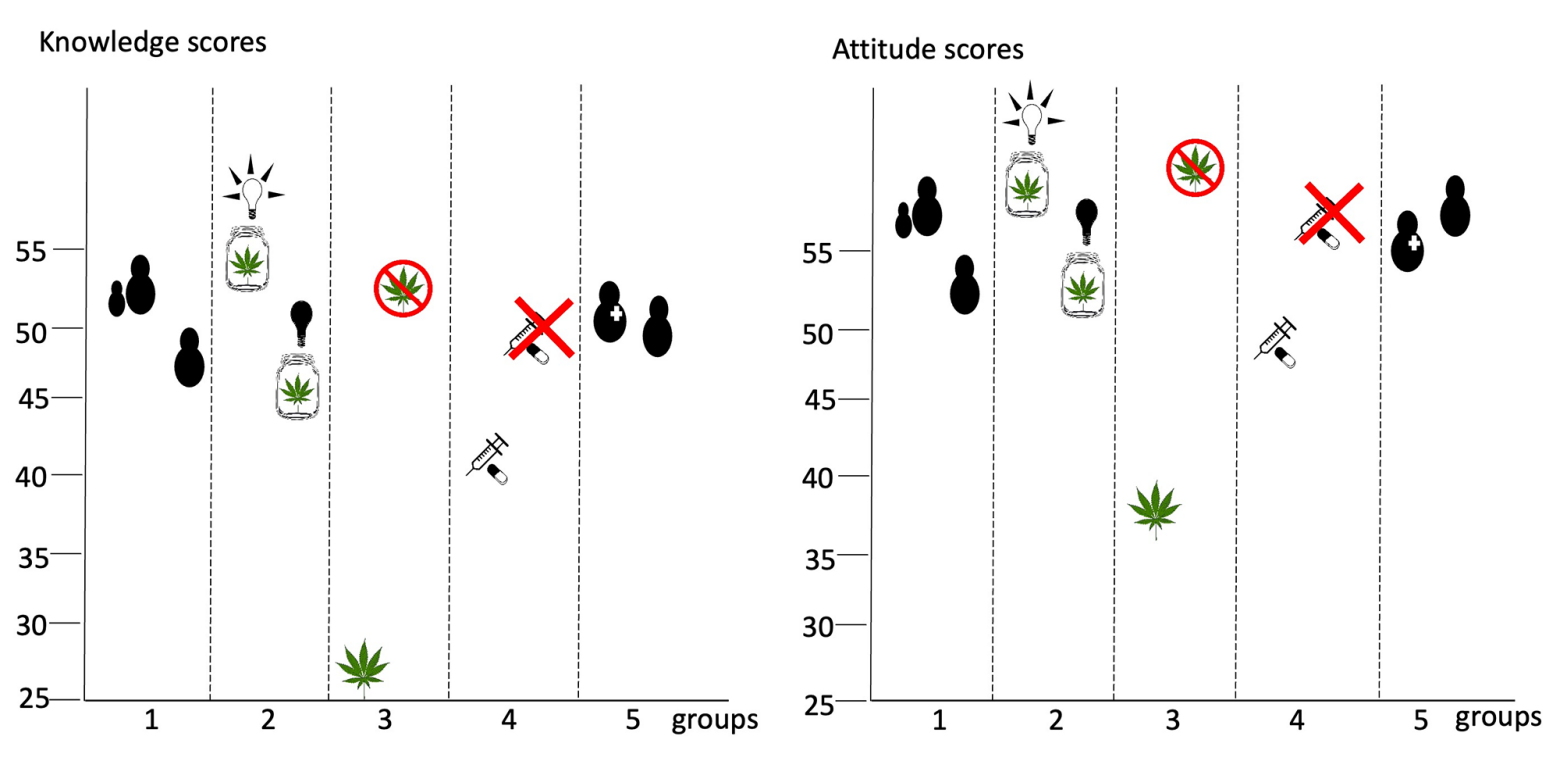
The survey on Knowledge and Attitudes of Legalizing Marijuana and differences in mean scores for knowledge and attitudes based on characteristics: (1) parents vs. non-parents; (2) knowing the definition of “medical marijuana” vs. not knowing; (3) not supporting marijuana legalization vs. supporting; (4) had not used controlled drugs without a doctor’s order vs. use of controlled drugs; and (5) medical/healthcare personnel vs. non-medical

The KALM survey data indicated the 38,502 respondents were knowledgeable about the facts related to marijuana and public health, social impacts, and medicine. However, respondents were much less knowledgable regarding the legal consequences of marijuana use. When we examined characteristics of respondents and scores on the KALM, those who were parents, religious, ≥ 36 years of age, had a high-income, did not smoke, drink or use controlled drugs without a prescription, knew there was no reliable evidence for the use of medical marijuana, and did not support marijuana legalization had higher scores for knowledge and attitudes (Fig. 1). Our findings are in contrast to studied in the US showing that counties with large college-educated populations and smaller percentages of individuals identifying as religious [28] and those with lower incomes [29] are more likely to approve of marijuana use.

We did not include therapeutic efficacy of marijuana in our survey because the data remains inconclusive and needs to be confirmed with studies that are randomized controlled trials using rigorous methodologies and large sample sizes [17]. Examining the therapeutic efficacy of marijuana is similar to the history of tobacco use, which was originally introduced in Europe as a treatment for disease at the end of the 15th century, became culturally acceptable globally as a social activity, and ultimately was identified as a causal factor for lung and oral cancer [30, 31].

Over 90% of respondents agreed that persistent cannabis use in adolescents would cause a loss of IQ points, which would not be restored by cessation of use. However, more than half of respondents were unaware of 1/3 of US high school students reported a lifetime of use of marijuana, even the use of marijuana was illegal for persons under 21 years of age. These findings echo those of a study reporting that parents in the state of Washington in the US were concerned with the possibility of increased risk of marijuana use in adolescents following legalization [32].

Data from the National Poison Data System (NPDS) showed that adolescent marijuana use increased 245% in the US from 2000 to 2020 [33]. This upward trend includes marijuana misuse/abuse in school-aged children and adolescents and reflects the impact of marijuana legalization on this vulnerable population [33]. Edibles became available in Canada in 2021 following national legalization and the proportion of marijuana-related emergency department visits with hospitalization for children aged 0–9 years increased significantly [4]. Although adolescents view marijuana as benign relative to other recreational drugs, marijuana use is linked to psychosis, suicidality, and cognitive impairment [34] and is known to impair sleep and the ability to drive a car [35].

Respondents who were medical and healthcare personnel had lower attitude scores and less concerns about marijuana than non-medical respondents. Although medical marijuana has been in use for cancer patients in the US, close to one-half of oncologists in one study did not recommend its’ clinical use, which might be explained by the fact that 70% did feel knowledgeable enough to make recommendations [36]. Thus, there is a need for more clinical trials exploring marijuana’s potential medicinal effects and the need for educational programs and incentivize trainings about medical marijuana for clinicians on this important issue.

Respondents who agreed with the statement there was no valid, reliable, or empirical evidence for “medical marijuana”, had significantly higher knowledge and attitude scores for all four categories of the KALM survey compared with those who disagreed with the statement. Although “medical marijuana” is legal for treating symptoms of illness in many states in the US, it is not FDA-approved [8]. A review of published medical marijuana studies conducted from 2000 to 2018 include pain management of multiple sclerosis, chronic pain, and cancer [37]. However, studies had mixed results, with most reporting an inability to draw conclusions due to inconsistent findings and a lack of rigorous evidence [37].

Scores for respondents who were supporters of legalizing marijuana were not only significantly lower compared with non-supporters but were also the lowest for knowledge or attitude among all other groups. These low scores indicate they were unaware of the harm associated with marijuana, which might suggest their support for legalization. Although the Marijuana Policy Project (MPP) [38] has suggested “Legalizing marijuana for adults has been a wise investment and provided states a new revenue stream to bolster budgets and fund important services and programs”, this has not been the actual case. Coloradans spend approximately $4.50 for every dollar spent on marijuana to mitigate the effects of legalization, with costs to the healthcare system stemming from accidental poisonings and traffic fatalities to increased court costs for impaired drivers, juvenile use, and employer-related costs [22].

Often, legalizing medical marijuana is considered a less expensive treatment for pain or psychological stress. However, Taiwan’s compulsory National Health Insurance (NHI) is available to everyone and used by most of the population. NHI provides comprehensive medical coverage, which allows users to seek medical treatment and without the concern about medical expenses. Taiwan’s FDA (TFDA) allows drugs containing CBD, but there are currently no domestically approved drugs with CBD and there is no rule or law allowing for the sale of CBD related products and drugs in Taiwan. If a doctor has prescribed drugs containing CBD, the patient may apply to import personal use quantities under “Regulations on Management of Medicament Samples and Gifts”. In addition, the American Heart Association issued a warning to help doctors and patients get better informed about the potentially harmful effects of marijuana use on brain health, especially in young and developing brains [39]. Therefore, there may be less urgency about legalizing marijuana in Taiwan.

## Conclusion

Over 38,000 respondents completed KALM survey; however, 104,000 clicked the link. This large number suggest that residents of Taiwan are concerned about the issue of marijuana. Determining if marijuana should be legalized in Taiwan requires knowledge about its effect on the public’s health and social impacts. Many respondents said more marijuana information should be provided to the public before Taiwan considers whether marijuana should be legalized. A clear definition is needed of the terms, “marijuana medications”, which are approved by the FDA or undergoing clinical trials, and “medical marijuana”, which has not been FDA-approved. The legalization of recreational marijuana also will require further input from the public of Taiwan. Therefore, we suggest information about the pros and cons of marijuana use be disseminated to the public via outreach programs and public health pamphlets.

### Limitations

This study had some limitations. First, this was an online survey, which cannot accurately describe the population to which it was distributed, and the respondents may be biased [40]. Second, although 85–90% of individuals over the age of 16 years have access to mobile phones and the Internet in Taiwan, those who do not have phones were unable to respond to the survey. Third, the complete rate of click of KALM was 38.4%, and this was indeed higher than the average click rates [41]. Therefore, clients who were familiar with/ interested in the topic “marijuana” may participate more in this online survey than those who are not. Fourth, though the distribution of respondents in different areas were similar to those in Taiwan, the risk-taking behavior of them had a low smoking rate and low alcohol consumption rate compared to the recent Taiwan prevalence rates on smoking and alcohol. Maybe people unreachable with the NGOs and academic societies could not participate if they did not know the link. Survey findings should be regarded with caution.

### Electronic supplementary material

Below is the link to the electronic supplementary material.

Supplementary Material 1

## Data Availability

The datasets generated and/or analyzed during the current study are available from the corresponding author on reasonable request.
