# Supplementary material for: Legalization of marijuana or not? Opinions from over 38,000 residents in Taiwan
Source: BMC Public Health. 2023 Oct 9;23:1954. doi: 10.1186/s12889-023-16834-x (PMC10563234; doi:10.1186/s12889-023-16834-x)
Supplement: Supplementary file 1 — Supplementary Material 1 [file 12889_2023_16834_MOESM1_ESM.docx]

**Supplementary Tables**

### Table S1 Residential city in Taiwan for respondents to the Knowledge and Attitude of Legalizing Marijuana survey (KALM) (N = 38, 502)

| Place of residence | n | % |  |
| --- | --- | --- | --- |
| Taipei City | | 8,658 | 22.5% |
| New Taipei City | | 8,223 | 21.4% |
| Keelung City | | 642 | 1.7% |
| Taoyuan City | | 3,428 | 8.9% |
| Hsinchu County | | 864 | 2.2% |
| Hsinchu City | | 929 | 2.4% |
| Miaoli County | | 443 | 1.2% |
| Taichung City | | 4,010 | 10.4% |
| Changhua County | | 886 | 2.3% |
| Nantou County | | 479 | 1.2% |
| Yunlin County | | 539 | 1.4% |
| Chiayi City | | 498 | 1.3% |
| Chiayi County | | 327 | 0.8% |
| Tainan City | | 2,006 | 5.2% |
| Kaohsiung City | | 4,107 | 10.7% |
| Pingtung County | | 625 | 1.6% |
| Yilan County | | 398 | 1.0% |
| Hualien County | | 571 | 1.5% |
| Taitung County | | 413 | 1.1% |
| Penghu County | | 72 | 0.2% |
| Kinmen County | | 93 | 0.2% |
| Other areas^†^ | | 291 | 0.8% |

^†^ Responses from those living outside of Taiwan who received the link from friends or relatives living in Taiwan.

### Table S2 Differences in mean scores for knowledge and attitude for the categories of public health, social impact, medicine and the use of marijuana, and legal and tax consequences on the Knowledge and Attitude of Legalizing Marijuana survey (KALM) (N = 38, 502)

|  | Knowledge |  |  | Attitudes |  |  |
| --- | --- | --- | --- | --- | --- | --- |
| Category/Characteristic | Mean (SD) | t | *p* | Mean (SD) | t | *p* |
| Public Health (range = 0 – 16 points) | |  |  |  |  |  |
| Parental status |  | 24.43 | **< 0.01** |  | 17.09 | **< 0.01** |
| Yes (n = 29,433) | 14.33 (2.93) |  |  | 14.95 (3.14) |  |  |
| No (n = 9,069) | 13.26 (3.85) |  |  | 14.21 (3.74) |  |  |
| Religious belief |  | 15.46 | **< 0.01** |  | 16.09 | **< 0.01** |
| Yes (n = 30,676) | 14.23 (3.03) |  |  | 14.93 (3.14) |  |  |
| No (n =7 ,826) | 13.52 (3.77) |  |  | 14.17 (3.86) |  |  |
| Age |  | -28.41 | **< 0.01** |  | -21.49 | **< 0.01** |
| ≤ 35 years (n = 4,198) | 12.31 (4.41) |  |  | 13.46 (4.31) |  |  |
| ≥ 36 years (n = 34,304) | 14.30 (2.95) |  |  | 14.93 (3.13) |  |  |
| Low/middle-low income |  | -15.40 | **< 0.01** |  | -3.8 | **< 0.01** |
| Yes (n =5,035) | 13.35 (3.69) |  |  | 14.60 (3.38) |  |  |
| No (n =33,467) | 14.19 (3.11) |  |  | 14.80 (3.30) |  |  |
| Cigarette smoker |  | -21.92 | **< 0.01** |  | -16.52 | **<0.01** |
| Yes (n =2,728) | 12.26 (4.59) |  |  | 13.45 (4.42) |  |  |
| No (n =35,774) | 14.22 (3.03) |  |  | 14.87 (3.19) |  |  |
| Alcohol consumption |  | -18.07 | **< 0.01** |  | -13.92 | **<0.01** |
| Yes (n =3,603) | 12.91 (4.19) |  |  | 13.88 (4.11) |  |  |
| No (n =34,899) | 14.20 (3.06) |  |  | 14.86 (3.20) |  |  |
| Used controlled drugs without a doctor’s order |  | -16.99 | **< 0.01** |  | -12.39 | **< 0.01** |
| Yes (n = 747) | 10.95 (5.12) |  |  | 12.69 (4.66) |  |  |
| No (n =37,755) | 14.14 (3.12) |  |  | 14.81 (3.26) |  |  |
| Medicine/healthcare personnel |  | 1.23 | 0.55 |  | -1.53 | **< 0.01** |
| Yes (n =2,622) | 14.16 (3.30) |  |  | 14.67 (3.44) |  |  |
| No (n =35,880) | 14.08 (3.20) |  |  | 14.78 (3.30) |  |  |
| There is no scientific support for medical marijuana |  | 36.07 | **< 0.01** |  | 23.17 | **< 0.01** |
| Agree (n = 16,604) | 14.77 (2.39) |  |  | 15.08 (3.11) |  |  |
| Disagree (n = 7,689) | 12.71 (4.75) |  |  | 13.86 (4.10) |  |  |
| Marijuana should be legalized |  | -62.27 | **< 0.01** |  | -47.06 | **< 0.01** |
| Agree (n = 1,970) | 7.45 (4.98) |  |  | 9.85 (4.88) |  |  |
| Disagree (n = 36,011) | 14.49 (2.60) |  |  | 15.08 (2.95) |  |  |
| Social Impact (range = 0 – 20 points) | |  |  |  |  |  |
| Parental status |  | 31.02 | **< 0.01** |  | 20.11 | **< 0.01** |
| Yes (n = 29,433) | 18.42 (3.90) |  |  | 18.75 (3.89) |  |  |
| No (n = 9,069) | 16.38 (5.87) |  |  | 17.61 (4.95) |  |  |
| Religious belief |  | 21.90 | **< 0.01** |  | 18.81 | **< 0.01** |
| Yes (n = 30,676) | 18.24 (4.10) |  |  | 18.71 (3.90) |  |  |
| No (n =7 ,826) | 16.73 (5.75) |  |  | 17.56 (5.09) |  |  |
| Age |  | -33.25 | **< 0.01** |  | -24.30 | **< 0.01** |
| ≤ 35 years (n = 4,198) | 14.68 (6.99) |  |  | 16.49 (5.81) |  |  |
| ≥ 36 years (n = 34,304) | 18.33 (3.94) |  |  | 18.72 (3.88) |  |  |
| Low/middle-low income |  | -12.36 | **< 0.01** |  | -5.04 | **< 0.01** |
| Yes (n =5,035) | 17.09 (5.30) |  |  | 18.19 (4.40) |  |  |
| No (n =33,467) | 18.06 (4.38) |  |  | 18.52 (4.16) |  |  |
| Cigarette smoker |  | -20.93 | **< 0.01** |  | -17.15 | **< 0.01** |
| Yes (n =2,728) | 15.26 (7.08) |  |  | 16.64 (5.91) |  |  |
| No (n =35,774) | 18.13 (4.20) |  |  | 18.62 (4.00) |  |  |
| Alcohol consumption |  | -19.17 | **< 0.01** |  | -15.79 | **< 0.01** |
| Yes (n =3,603) | 16.05 (6.38) |  |  | 17.14 (5.45) |  |  |
| No (n =34,899) | 18.13 (4.24) |  |  | 18.61 (4.02) |  |  |
| Used controlled drugs without a doctor’s order |  | -18.66 | **< 0.01** |  | -13.20 | **< 0.01** |
| Yes (n = 747) | 12.54 (8.03) |  |  | 15.43 (6.41) |  |  |
| No (n =37,755) | 18.04 (4.36) |  |  | 18.54 (4.12) |  |  |
| Medicine/healthcare personnel |  | -2.11 | **< 0.01** |  | -2.42 | **< 0.01** |
| Yes (n =2,622) | 17.74 (5.00) |  |  | 18.28 (4.48) |  |  |
| No (n =35,880) | 17.95 (4.49) |  |  | 18.49 (4.17) |  |  |
| There is no scientific support for medical marijuana |  | 50.51 | **< 0.01** |  | 27.44 | **<0.01** |
| Agree (n = 16,604) | 19.58 (1.96) |  |  | 19.00 (3.73) |  |  |
| Disagree (n = 7,689) | 15.24 (7.41) |  |  | 17.10 (5.53) |  |  |
| Marijuana should be legalized |  | -83.57 | **< 0.01** |  | -49.96 | **<0.01** |
| Agree (n = 1,970) | 5.92 (6.74) |  |  | 11.20 (6.82) |  |  |
| Disagree (n = 36,011) | 18.68 (3.17) |  |  | 18.94 (3.55) |  |  |
| Medicine and use of THC (range = 0 – 12 points) | | | | | | |
| Parental status |  | 11.02 | **< 0.01** |  | 16.87 | **< 0.01** |
| Yes (n = 29,433) | 9.73 (2.34) |  |  | 11.08 (2.28) |  |  |
| No (n = 9,069) | 9.40 (2.59) |  |  | 10.56 (2.67) |  |  |
| Religious belief |  | 7.65 | **< 0.01** |  | 17.85 | **<0.01** |
| Yes (n = 30,676) | 9.70 (2.37) |  |  | 11.08 (2.26) |  |  |
| No (n =7 ,826) | 9.46 (2.53) |  |  | 10.48 (2.79) |  |  |
| Age |  | -14.83 | **<0.01** |  | -21.86 | **<0.01** |
| ≤ 35 years (n = 4,198) | 9.05 (2.86) |  |  | 10.01 (3.05) |  |  |
| ≥ 36 years (n = 34,304) | 9.73 (2.34) |  |  | 11.08 (2.26) |  |  |
| Low and middle-low income |  | -13.05 | **<0.01** |  | -4.01 | **<0.01** |
| Yes (n =5,035) | 9.22 (2.54) |  |  | 10.83 (2.48) |  |  |
| No (n =33,467) | 9.70 (2.38) |  |  | 10.98 (2.37) |  |  |
| Cigarette smoker |  | -16.19 | **< 0.01** |  | -15.45 | **<0.01** |
| Yes (n =2,728) | 8.81 (2.85) |  |  | 10.08 (3.15) |  |  |
| No (n =35,774) | 9.72 (2.36) |  |  | 11.03 (2.30) |  |  |
| Alcohol consumption |  | -13.12 | **< 0.01** |  | -15.01 | **< 0.01** |
| Yes (n =3,603) | 9.09 (2.74) |  |  | 10.27 (2.93) |  |  |
| No (n =34,899) | 9.71 (2.36) |  |  | 11.03 (2.31) |  |  |
| Used controlled drugs without a doctor’s order |  | -12.89 | **< 0.01** |  | -12.16 | **< 0.01** |
| Yes (n = 747) | 8.29 (2.94) |  |  | 9.47 (3.40) |  |  |
| No (n =37,755) | 9.68 (2.39) |  |  | 10.99 (2.35) |  |  |
| Medicine/healthcare personnel |  | 2.09 | 0.31 |  | -1.92 | **< 0.01** |
| Yes (n =2,622) | 9.75 (2.45) |  |  | 10.87 (2.47) |  |  |
| No (n =35,880) | 9.65 (2.40) |  |  | 10.97 (2.38) |  |  |
| There is no scientific support for medical marijuana |  | 42.31 | **< 0.01** |  | 26.41 | **< 0.01** |
| Agree (n = 16,604) | 10.37 (2.17) |  |  | 11.31 (2.25) |  |  |
| Disagree (n = 7,689) | 8.81 (2.88) |  |  | 10.33 (2.86) |  |  |
| Marijuana should be legalized |  | -39.98 | **< 0.01** |  | -41.84 | **< 0.01** |
| Agree (n = 1,970) | 6.91 (3.19) |  |  | 7.80 (3.53) |  |  |
| Disagree (n = 36,011) | 9.83 (2.25) |  |  | 11.17 (2.15) |  |  |
| Legal and Tax consequences (range = 0 – 12 points) | | | | | | |
| Parental status |  | 17.82 | **< 0.01** |  | 18.27 | **< 0.01** |
| Yes (n = 29,433) | 8.14 (3.13) |  |  | 11.07 (2.29) |  |  |
| No (n = 9,069) | 7.45 (3.27) |  |  | 10.49 (2.76) |  |  |
| Religious belief |  | 11.83 | **< 0.01** |  | 18.48 | **< 0.01** |
| Yes (n = 30,676) | 8.08 (3.15) |  |  | 11.07 (2.28) |  |  |
| No (n =7 ,826) | 7.59 (3.26) |  |  | 10.42 (2.86) |  |  |
| Age |  | -16.53 | **< 0.01** |  | -23.07 | **< 0.01** |
| ≤ 35 years (n = 4,198) | 7.17 (3.37) |  |  | 9.90 (3.16) |  |  |
| ≥ 36 years (n = 34,304) | 8.08 (3.14) |  |  | 11.06 (2.29) |  |  |
| Low and middle-low income |  | -11.65 | **< 0.01** |  | -5.14 | **< 0.01** |
| Yes (n =5,035) | 7.49 (3.19) |  |  | 10.77 (2.53) |  |  |
| No (n =33,467) | 8.05 (3.17) |  |  | 10.96 (2.41) |  |  |
| Cigarette smoker |  | -9.88 | **< 0.01** |  | -16.76 | **< 0.01** |
| Yes (n =2,728) | 7.36 (3.41) |  |  | 9.95 (3.24) |  |  |
| No (n =35,774) | 8.02 (3.16) |  |  | 11.01 (2.33) |  |  |
| Alcohol consumption |  | -8.83 | **< 0.01** |  | -15.34 | **< 0.01** |
| Yes (n =3,603) | 7.51 (3.37) |  |  | 10.22 (3.01) |  |  |
| No (n =34,899) | 8.03 (3.15) |  |  | 11.01 (2.34) |  |  |
| Use of controlled drugs without a doctor’s order |  | -8.37 | **<0.01** |  | -12.81 | **<0.01** |
| Yes (n = 747) | 6.95 (3.41) |  |  | 9.35 (3.43) |  |  |
| No (n =37,755) | 7.80 (3.17) |  |  | 10.97 (2.39) |  |  |
| Medicine/healthcare personnel |  | 5.34 | **< 0.01** |  | -1.78 | **< 0.01** |
| Yes (n =2,622) | 8.31 (3.28) |  |  | 10.85 (2.59) |  |  |
| No (n =35,880) | 7.95 (3.17) |  |  | 10.94 (2.41) |  |  |
| There is no scientific support for medical marijuana |  | 34.47 | **< 0.01** |  | 28.13 | **< 0.01** |
| Agree (n = 16,604) | 9.13 (2.93) |  |  | 11.30 (2.23) |  |  |
| Disagree (n = 7,689) | 7.58 (3.42) |  |  | 10.22 (2.98) |  |  |
| Marijuana should be legalized |  | -45.32 | **<0.01** |  | -48.33 | **< 0.01** |
| Agree (n = 1,970) | 4.92 (3.12) |  |  | 7.28 (3.54) |  |  |
| Disagree (n = 36,011) | 8.19 (3.08) |  |  | 11.17 (2.15) |  |  |

Note: SD = standard deviation; THC = Δ⁹-tetrahydrocannabinol

### Table S3 Differences in mean total scores (range = 0-60 points) for knowledge and attitude for the categories of public health, social impact, medicine and the use of marijuana, and legal and tax consequences on the Knowledge and Attitude of Legalizing Marijuana survey (KALM)

|  | Knowledge |  |  | Attitude |  |  |
| --- | --- | --- | --- | --- | --- | --- |
| Characteristic | Mean (SD) | t | *p* | Mean (SD) | t | *p* |
| Parental status |  | 28.86 | **< 0.01** |  | 19.22 | **<0.01** |
| Yes (n = 29,433) | 50.62 (9.28) |  |  | 55.85 (11.10) |  |  |
| No (n = 9,069) | 46.48 (12.66) |  |  | 52.86 (13.47) |  |  |
| Religious belief |  | 19.50 | **< 0.01** |  | 18.65 | **< 0.01** |
| Yes (n = 30,676) | 50.25 (9.62) |  |  | 55.79 (11.05) |  |  |
| No (n =7 ,826) | 47.30 (12.47) |  |  | 52.62 (13.96) |  |  |
| Age |  | -31.22 | **< 0.01** |  | -24.06 | **< 0.01** |
| ≤ 35 years (n = 4,198) | 43.21 (14.64) |  |  | 49.86 (15.51) |  |  |
| ≥ 36 years (n = 34,304) | 50.44 (9.37) |  |  | 55.80 (11.05) |  |  |
| Low/middle-low income |  | -16.68 | **< 0.01** |  | -4.75 | **< 0.01** |
| Yes (n =5,035) | 47.16 (11.56) |  |  | 54.39 (12.21) |  |  |
| No (n =33,467) | 50.02 (10.08) |  |  | 55.26 (11.70) |  |  |
| Cigarette smoker |  | -21.90 | **< 0.01** |  | -17.37 | **< 0.01** |
| Yes (n =2,728) | 43.70 (15.04) |  |  | 50.12 (15.97) |  |  |
| No (n =35,774) | 50.10 (9.73) |  |  | 55.53 (11.30) |  |  |
| Alcohol consumption |  | -19.17 | **< 0.01** |  | -15.77 | **< 0.01** |
| Yes (n =3,603) | 45.56 (13.79) |  |  | 51.52 (14.81) |  |  |
| No (n =34,899) | 50.07 (9.81) |  |  | 55.52 (11.34) |  |  |
| Use of controlled drugs without a doctor’s order |  | -18.37 | **< 0.01** |  | -13.45 | **< 0.01** |
| Yes (n = 747) | 38.73 (16.52) |  |  | 46.94 (16.93) |  |  |
| No (n =37,755) | 49.87 (10.05) |  |  | 55.31 (11.59) |  |  |
| Medicine/healthcare personnel |  | 1.45 | **< 0.01** |  | -2.05 | **< 0.01** |
| Yes (n =2,622) | 49.95 (11.06) |  |  | 54.67 (12.39) |  |  |
| No (n =35,880) | 49.63 (10.27) |  |  | 55.18 (11.72) |  |  |
| There is no scientific support for medical marijuana |  | 51.91 | **< 0.01** |  | 27.52 | **< 0.01** |
| Agree (n = 16,604) | 53.86 (6.22) |  |  | 56.69 (10.95) |  |  |
| Disagree (n = 7,689) | 44.33 (15.51) |  |  | 51.51 (14.70) |  |  |
| Marijuana should be legalized |  | -80.40 | **< 0.01** |  | -51.58 | **< 0.01** |
| Agree (n = 1,970) | 25.20 (14.22) |  |  | 36.14 (17.23) |  |  |
| Disagree (n = 36,011) | 51.19 (7.99) |  |  | 56.36 (10.31) |  |  |

Note: SD = standard deviation

**Table S4** Examples of personal feedback from the open-ended survey question

| Respondent | Age group | Region of Taiwan or other countries | Topic and comments |
| --- | --- | --- | --- |
|  |  |  | **Promote accurate information about marijuana** |
| Mother | 46-55 years | Taipei | The government should educate the public about other countries’ experiences of legalization of marijuana |
| Non-parent, male | 36-45 years | Taipei | Information on marijuana use is important, but it has not been provided. There are huge problems, which can be disasters if marijuana is legalized without information about these concerns. |
| Mother | ≥ 56 years | Taipei | The government and non-profit organization should hire experts to teach students and parents about the problems of drugs and marijuana. |
|  |  |  | **Personal experiences with and reactions to marijuana** |
| Non-parent, male | ≥ 56 years | Taipei | I have smoked marijuana several times in the United States, and concluded it was not a good thing to do. I feel it must be regulated, and it should not be used arbitrarily. |
| Mother | 46-55 years | Kee-Lung | My son is an addict and has suffered greatly from using marijuana. He is under addiction treatment. I strongly oppose the legalization of marijuana in Taiwan. |
| Non-parent, female | 46-55 years | Taipei | My nephew developed a habit of smoking marijuana in the United States. He became restless, lost his appetite, and relied on drinking to relieve depression when he came back to Taiwan. He neglected his studies. Marijuana can be addictive. |
| Non-parent, female | ≥ 56 years | Unknown | I witnessed my friend become addicted to marijuana. He wanted more drugs, became paranoid, and could no longer work as a skilled technician. |
|  |  |  | **Use of marijuana abroad in regions where it is legal** |
| Mother | 46-55 years | Seattle | I live in Seattle where marijuana is legal and have seen the physical and societal harm it can cause. I sincerely hope that Taiwan will not follow in their footsteps. |
| Mother | 46-55 years | USA | I am Taiwanese with a child in middle school in the United States. Recently, I have regretted having supported legalization of marijuana because it caused my child harm. I hope people will not let marijuana use become legal in Taiwan. |
| Mother | ≥ 56 years | New Taipei | When I was living in the United States, a group of children and parents were waiting on the side of the road after singing at church on Christmas Eve. A middle-aged lady, who had been smoking marijuana, drove into the crowd, killing six people and seriously injuring more than a dozen. So telling people that marijuana is just a soothing and harmless substance is narrow-minded and uninformed. |
|  |  |  | **Controversy of ‘medical’ versus ‘recreational’ marijuana and pharmaceutical standards** |
| Mother | 46-55 years | Tainan | It is okay to legalize ‘medical marijuana’, but ‘recreational marijuana’ should not be legalized. |
| Non-parent, male | ≥ 56 years | New Taipei | The question is to distinguish CBD or THC. I support legalizing CBD but not THC in Taiwan. |
| Mother | 46-55 years | Taipei | Medical marijuana is a fraud. Calling dried marijuana plants ‘medical marijuana’ is like sailing under false colors. It triggers confusion among patients, nurses, the public, and even physicians. We want FDA approved and regulated marijuana medications, rather than the herb-like “medical marijuana”. |
| Father | ≥ 56 years | New Taipei | Criminals and unlawful businessmen are mainly interested in profit; they disguise the illegality in the name of “medical marijuana”. The legislators or public officials should not legalize marijuana by vote. |
|  |  |  | **Economic reasons for not legalizing marijuana (harms outweigh benefits)** |
| Mother | 25-35 years | Hualien County | The government sells tobacco and alcohol, and then uses tax money to promote quitting smoking and drinking. It is like putting the cart before the horse. We should not repeat the same mistakes with marijuana! |
| Mother | ≥ 56 years | Taipei | If the government lists marijuana as an important part of the economy, the government is simply short-sighted. |
|  |  |  | **Support for legalization of marijuana** |
| Non-parent, male | 25-35 years | Taipei | Marijuana is legalized in many European countries, United States and Thailand; it is progressive. |
| Father | 46-55 years | Yilan County | You don’t understand marijuana. Marijuana can treat many diseases. |
